# Nicotinic cholinergic system and COVID-19: *In silico* evaluation of nicotinic acetylcholine receptor agonists as potential therapeutic interventions

**DOI:** 10.1016/j.toxrep.2020.12.013

**Published:** 2020-12-19

**Authors:** Nikolaos Alexandris, George Lagoumintzis, Christos T. Chasapis, Demetres D. Leonidas, Georgios E. Papadopoulos, Socrates J. Tzartos, Aristidis Tsatsakis, Elias Eliopoulos, Konstantinos Poulas, Konstantinos Farsalinos

**Affiliations:** a Laboratory of Molecular Biology and Immunology, Department of Pharmacy, University of Patras, 26500, Rio-Patras, Greece; b Institute of Research and Innovation - IRIS, Patras Science Park SA, 26500 Patras, Greece; c Department of Biochemistry and Biotechnology, University of Thessaly, Biopolis, 41500 Larissa, Greece; d Tzartos NeuroDiagnostics, 3, Eslin Street, Athens 115 23, Greece; e Department Medicine, University of Crete, Greece; f Department of Biotechnology, Laboratory of Genetics, Agricultural University of Athens, Iera Odos 75, 11855 Athens, Greece

**Keywords:** ACh, Acetylcholine, AChBP, Acetylcholine-binding protein, ARDS, acute respiratory distress syndrome, BLAST, Basic Local Alignment Search Tool, CHARMM, Chemistry at Harvard Macromolecular Mechanics, CNS, Central Nervous System, CoV, coronavirus, DCD, single precision binary FORTRAN, ECD, extracellular domain, HADDOCK, High Ambiguity Driven protein-protein DOCKing, HMGB1, High-mobility group protein 1, IL, Interleukin, Jak2, Janus kinases 2, STAT3, signal transducer and activator of transcription 3, LBD, Ligand Binding Domain, lig, ligand, MD, Molecular Dynamics, MDS, Molecular Dynamics Simulations, MERS, Middle East Respiratory Syndrome, nAChRs, nicotinic acetylcholine receptors, NAMD, Nanoscale Molecular Dynamics, NCBI, National Center for Biotechnology Information, NCS, Nicotinic Cholinergic System, NF-kB, nuclear factor kappa-light-chain-enhancer of activated B cells, NPT, constant number, pressure, energy, NVT, constant number, volume, energy, PDB, Protein Data Bank, PME, Particle Mesh Ewald, PRODIGY, PROtein binDIng enerGY prediction, PyMOL, Python Molecule, RBD, Receptor Binding Domain, RMSD, Root-mean-square deviation, SARS, Severe Acute Respiratory Syndrome, SARS-CoV-2 S1, SARS - 2 Spike Subunit 1 protein, STD NMR, Saturation Transfer Difference Nuclear Magnetic Resonance, TNF, Tumor Necrosis Factor, VMD, Visual Molecular Dynamics, Cholinergic agonists, COVID-19, Nicotinic acetylcholine receptors, SARS-CoV-2, Spike glycoprotein

## Abstract

SARS-CoV-2 infection was announced as a pandemic in March 2020. Since then, several scientists have focused on the low prevalence of smokers among hospitalized COVID-19 patients. These findings led to our hypothesis that the Nicotinic Cholinergic System (NCS) plays a crucial role in the manifestation of COVID-19 and its severe symptoms. Molecular modeling revealed that the SARS-CoV-2 Spike glycoprotein might bind to nicotinic acetylcholine receptors (nAChRs) through a cryptic epitope homologous to snake toxins, substrates well documented and known for their affinity to the nAChRs. This binding model could provide logical explanations for the acute inflammatory disorder in patients with COVID-19, which may be linked to severe dysregulation of NCS. In this study, we present a series of complexes with cholinergic agonists that can potentially prevent SARS-CoV-2 Spike glycoprotein from binding to nAChRs, avoiding dysregulation of the NCS and moderating the symptoms and clinical manifestations of COVID-19. If our hypothesis is verified by *in vitro* and *in vivo* studies, repurposing agents currently approved for smoking cessation and neurological conditions could provide the scientific community with a therapeutic option in severe COVID-19.

## Highlights

• SARS-CoV-2 could interact with nAChRs triggering Nicotinic Cholinergic anti-inflammatory system dysregulation.
• The α7 nAChRs and SARS-CoV-2 S1 interaction is significantly disturbed by the binding of AChRs agonists.
• AChRs may be an intriguing therapeutic approach for the COVID-19s' pandemic.

## Graphical abstract

**Figure:**
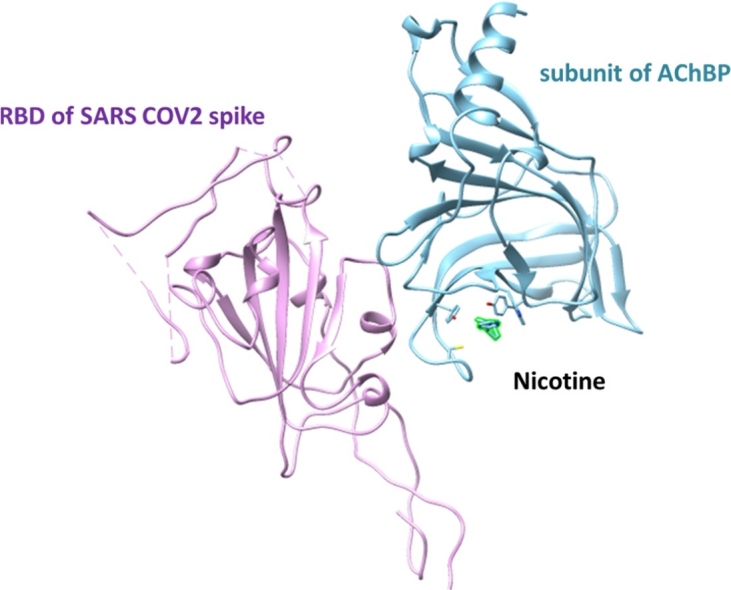

## 1 Introduction

Recent epidemiological research has established a clear correlation between long-term exposure to synthetic anthropogenic chemicals and the production of chronic diseases and environmental pollution. Environmental chemicals suppress or impair immune response and effectiveness through immunomodulation signaling pathways, making individuals more vulnerable to new viral epidemics/pandemics, such as coronavirus respiratory diseases [1].

Coronaviruses are a broad family of enveloped RNA viruses that can infect humans and animals, causing respiratory and enteric diseases. The first lethal Severe Acute Respiratory Syndrome (SARS)-causing coronavirus (CoV) occurred in 2002 in China, with approximately 800 people succumbing, while a few years later a second was identified in the Middle East, dubbed the Middle East Respiratory Syndrome (MERS)-CoV, this time with a death toll of up to 858 people [2]. SARS-CoV-2 infection represents the third coronavirus that has caused global alarm, after SARS-CoV and MERS-CoV. However, the latest strain has exceeded previous strains' global health implications and was declared a pandemic in March 2020 (named Corona Virus Disease 2019, COVID*-19),* and has claimed more than 1 million lives until now [3].

SARS-CoV-2 poses a clear and present risk, with factors such as bias, state structure, decisive and actionable behavior playing a pivotal role in solving the problem while the scientific community is agonizingly trying to find a medical solution. Several factors such as lifestyle, iatrogenic or environmental factors may lead to an increase in viral epidemics. These factors may have a rapid and substantial effect on infectious epidemiological curves, contributing to a sharp increase in the outbreak that continues to occur in epidemic waves. Administrative problems, human bias, and bureaucracy tend to be critical factors for extreme outbreaks [4].

Symptoms of SARS-CoV-2 infection typically occur after an incubation period of approximately five days (from 3 to 14 days). In deceased patients, the time from onset to death varies from 6 to 41 days, with a median of 14 days [[5], [6], [7]]. Most SARS-CoV-2 infected patients have mild symptoms, including dry cough, sore throat, fever, muscle, bony aches, and spontaneous shortness of breath. Comorbid adults and elderly subjects experience multiple complications, including bilateral pulmonary infiltration of chest imaging, leading to severe pneumonia, septic shock, pulmonary edema, and acute respiratory distress syndrome (ARDS), despite invasive ventilation. The epidemiological data for patients include several varying clinical effects. Respiratory manifestations are responsible for the severity of the disease, whereas the impact of COVID-19 on kidneys is frequently observed in positively tested subjects [8]. Neurological complications present in patients are not decidedly clear since their expression is either because of a general inflammatory effect on the CNS or a direct effect of the viral-induced neural impairment. In contrast, immunosuppressed individuals with COVID-19 are under severe threat and should partake in their treatment's specific dosage [9]. The cardiovascular complications are present and have similarities with SARS CoV, MERS, and influenza since there appear to have a strong resemblance [10]. The manifestation of cytokine storm in conjunction with myocardial injury, development of cardiomyopathy without respiratory symptoms, unspecified arrhythmia contribute massively to mortality and warrant special treatment in the form of anticoagulant care, ACE inhibitors and angiotensin receptor blockers [8,10]. Generally, numerous clinical manifestations are present in COVID-19 patients, such as encephalopathy, encephalitis, seizures, cerebrovascular events, gastrointestinal, liver manifestations, anosmia, and ageusia [[8], [9], [10], [11]].

To date, there are no broadly proven successful therapies for COVID-19, although some therapies have demonstrated certain benefits in particular patient subpopulations or for specific endpoints. Physicians and researchers based their attention on the repurposing of existing drugs to tackle COVID-19 severe cases as preventive measures such as social isolation and mask use were both psychologically and economically expensive [12,13]. Still, the most awaited means against the vaccine, COVID-19, is the focus of various laboratories, continually investigating and exploring the methods to be used, challenged by its clinical safety and efficacy, as well as the time-critical nature of its testing and development [[12], [13], [14]].

Efficient viral invasion and replication can lead to an aggressive hyperinflammatory immune response, which compromises immune homeostasis [15,16] with the release of a large number of proinflammatory cytokines, a clinical event referred to as the "cytokine storm" (CS) [17]. CS is characterized by a clinical appearance of overwhelming systemic inflammation, hyperferritinemia, hemodynamic dysfunction, and multi-organ failure that can lead to death. The cause for CS is an unregulated immune response, resulting in continuous activation and expansion of immune cells, lymphocytes, and macrophages, which generate vast quantities of cytokines, resulting in a cytokine storm. CS clinical results are due to the action of proinflammatory cytokines such as IL-1, IL-18, IFN-γ, IL-6, and TNF-α, with the latter being more critical contributors [18,19]. Early detection of CS and prompt treatment can lead to a better outcome. Several biological agents targeting cytokines have been suggested for CS treatment. Cytokine storm appears to be one of the common causes of death in the newly declared COVID-19 pandemic. Therapeutic approaches to managing the COVID-19 cytokine storm can provide an opportunity to reduce the morbidity and mortality associated with COVID-19.

An increasing body of evidence also indicates that COVID-19 may not be confined to the respiratory tract, as SARS-CoV-2 may invade the central nervous system (CNS) [9,20]. Acetylcholine (Ach) and its nicotinic receptors (*i.e.*, nicotinic acetylcholine receptors, nAChRs) are one of the most important CNS neurotransmitters, and the cholinergic pathway plays an important role in modulating inflammatory response [21]. Among the various nicotinic receptor subtypes, the α7 receptor is the most important mediator of the anti-inflammatory properties of the cholinergic system due to its high expression in immune cells (B cells, macrophages, T cells, and macrophages) and its association with humoral and intrinsic immunity [22]. Physiological (secreted ACh) or pharmacological (exposure to agonists) stimulation of homopentameric α7 nAChR, present on the surface of tissue macrophages, is well known to block proinflammatory cytokine expression (*i.e.*, TNF-α, IL-1, IL-6) [[23], [24], [25]]. Remarkably, the α7 nAChR controls cytokine production at the post-transcriptional level without affecting levels of mRNAs for TNF-α, IL-1, IL-6, and IL-18. The scenario is different for HMGB1, the constitutive intracellular expression vital for macrophages' survival and normal transcriptional regulation [26]. Instead of HMGB1 translation, α7 nAChR prevents secretion, most likely by inhibiting its translocation to the cytoplasm [24] from the nucleus. Numerous studies have demonstrated the inhibitory α7-mediated action of nicotine in the nuclear factor-kB (NF-kB) pathway necessary to activate macrophage and proinflammatory cytokine secretion [24,27,28]. The Jak2-STAT3 pathway is likewise involved, as shown both *in vivo* and *in vitro* since α7 nAChR stimulation after nicotine binding causes phosphorylation of Jak2, which triggers STAT3 [29]. Besides their effect on the immune system, a7 nAChRs are expressed in other cells such as lymphocytes, monocytes, macrophages, dendritic cells, adipocytes, keratinocytes, endothelial cells, and epithelial cells of the intestine and lung [[30], [31], [32], [33]]. Therefore, nAChRs could be implicated in the pathophysiology of severe COVID-19 *via* mechanisms independent of the cholinergic anti-inflammatory pathway [11]. In any case, evaluating pharmacological approaches to activate α7 nAChR to inhibit immunologic phenomena such as cytokine storm or prevent clinical manifestations of severe COVID-19 may have some clinical value.

Observation of the low prevalence of hospitalized COVID-19 patients in China has led to the hypothesis that nicotine may have protective effects by stimulating the cholinergic anti-inflammatory pathway [11,34]. Additionally, it has been postulated that parts of SARS-CoV-2 S1 protein may bind to nAChRs and adversely affect their function by preventing acetylcholine's binding and action. In this context, we recently suggested that the NCS might be involved in the pathophysiology of COVID-19. We also extended this hypothesis by studying *in silico* molecular interactions between the human neuronal α7 and the spike glycoprotein of SARS-CoV-2 [35,36]. Expanding our previously published work, we present the *in silico* molecular interactions of the protein complexes between the homologous to the extracellular domain (ECD) of the human α7 AChR, the acetylcholine binding protein (AChBP) bound to the most known nAChRs' agonists, and the SARS-CoV-2 Spike glycoprotein. Moreover, we present the docking of the endogenous agonist acetylcholine, as well as of several other exogenous agonists like nicotine [37], carbamylcholine [38], galantamine [39], epibatidine [40], varenicline [41], succinylcholine [42], and cytisine [43], to the S1- α7 nAChR complex [35], to highlight their pharmacologic potential against COVID-19.

## 2 Methods

### 2.1 Sequence retrieval and alignment

Sequence retrieval and alignment between SARS-CoV's Spike glycoproteins have been previously shown by our group [35,36]. Likewise, sequence alignment (of nAChRs, AChBP, and coordinating residues of cholinergic agonists studied) was performed using the National Center for Biotechnology Information databases (USA), the Mega BLAST with the UniProt protein database, and the BLASTP (protein-protein BLAST) with default parameters.

### 2.2 Structure retrieval

We used the following 3D structures for our purposes, all downloaded from the Protein Data Bank: i) the SARS-CoV-2 Spike glycoprotein complexed with the human ACE2 (PDB id: 6LZG), ii) the *Lymnaea stagnalis* or *Aplysia californica* AChBP complexed with nicotine (PDB id: 1UW6), with cytisine (PDB id: 4BQT), and with varenicline (PDB id: 4AFT), iii) the human α3β4 nAChR with α-conotoxin (PDB id: 5T90), iv) the structure of epibatidine (PDB id: 3SQ6), and v) the structure of the ligand-binding domain (LBD) of a chimera pentameric α7 nAChR (PDB id: 3SQ9).

**Fig. 1:**
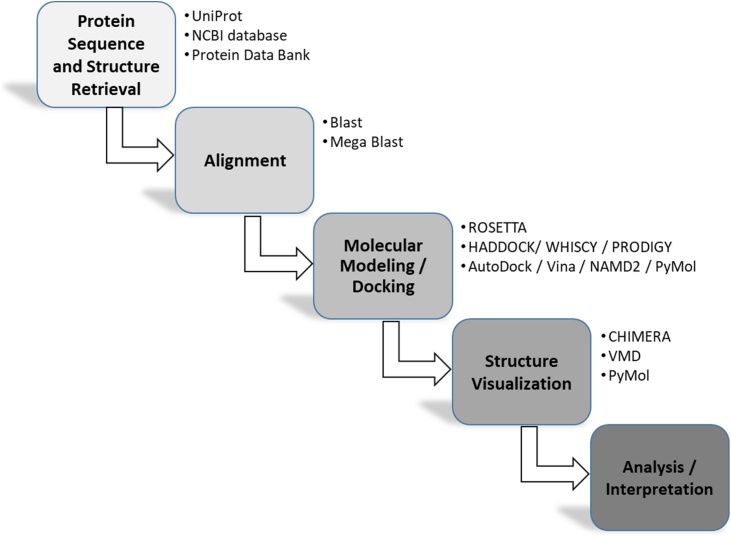
Schematic workflow of our in-silico approach.

Fig. 1 displays the schematic workflow demonstrating our methodology.

### 2.3 Molecular modeling and docking experiments

Using ROSETTA, the protein structure prediction of human ECD of α7 nAChR was performed with an automated multi-step and multi-template homology modeling approach [44]. Complexes between SARS-CoV-2 Spike glycoprotein with AChBP -corresponding to the homologous ECD of the human pentameric α7 nAChR- bound to the nAChRs potential interacting agonists were modeled using the HADDOCK server [67]. WHISCY software automatically created the expected interaction surfaces and the ambiguous interaction restraints (AIRs) used for driving the HADDOCK process.

Previously experimentally defined conserved residues involved in the interaction between α7 nAChR chimera and α-bungarotoxin [45] were used in input the WHISCY program to predict the interaction surface between ECD of AChBP and SARS-CoV-2 Spike glycoprotein. PRODIGY [46] software was used to predict the binding affinity of biomolecular complexes [46], while UCSF Chimera software was used to visualize all protein structures [47].

The complex structure of the pentameric α7 nAChR with SARS-CoV-2 Spike (S1) glycoprotein presented recently [35] was used for the docking of the agonists. In a first step, the complex of S1 with its two interacting α7 chains (A, B) of the α7 nAChR pentamer was isolated from the initial complex and energy minimized using the Molecular Dynamics (MD) program NAMD2 [48] with topology and parameters of the CHARMM force field for proteins (top_all36_prot.rtf, par_all36m_prot.prm). For this work, we will refer to this energy minimized trimer as the S1-2α7 complex. The ligands' structures were positioned in the S1-2α7 complex by superposing their corresponding protein subunits using PyMol [49]. In order to remove clashes of some ligands with amino acid residues in their new environment, we repositioned them according to the results of a rigid body (only ligand flexible) docking using the program AutoDock Vina [50] with grid box size 16 × 16 × 16, spacing 1 Å and exhaustiveness 16. Ligand models with the minimum difference from their original position and orientation were selected for further modeling. In the same step, for each ligand, a set of S1-2α7 residues were defined as belonging to the binding pocket if their atoms were in a distance ≤ 5.0 Å from ligand atoms. These sets of residues (not identical for all ligands) have been set as flexible in additional docking studies. The grid box size was 20 × 20 × 20, with a spacing of 1 Å and exhaustiveness 16. Using the models resulted from the flexible docking study, we prepared for each ligand a complex with S1-2α7 named S1-2α7-lig (chains F, A, B and L), where "lig" represents each member of the ligand series [acetylcholine (ach), nicotine (nct), carbamylcholine (cce), galantamine (gnt), epibatidine (epj), varenicline (qmr), succinylcholine (sck), cytisine (c53]. We have also included in the modeling procedure the four disulphide bond pairs found in S1 (^336−361^F, ^379-432^F, ^91-525^F, ^480-488^F) and one pair for each of chain A and B (^125^A - ^138^A, ^125^B - ^138^B). These complexes were then hydrated with 25715 water molecules. Charge neutrality has been obtained by adding 73 Na^+^ and 16 Cl^―^ ions reaching a salt concentration of 0.15 mol/L. Before starting Molecular Dynamics Simulations (MDS), we first energy minimized each one of the so prepared systems with constrained C_α_ atoms and ligand atoms using topology and parameters as described above. Topology and parameters for the ligands were taken from SwissParam [51]. Next, for each of the energy minimized systems, a short NPT simulation was run at P = 1 Atm to gradually increase the temperature from 0 to 300 K and stabilize the simulation cell dimensions. During these simulations, S1 atoms were free to move, while harmonic constraints were applied to the backbone atoms of the 2α7 dimer, with ligand atoms fixed.

Moreover, Periodic Boundary Conditions and the PME (Particle Mesh Ewald) algorithm for handling electrostatics were applied. The NPT simulations were followed by 20 ns NVT simulations at 300 K keeping all other conditions the same as before. To reduce the file size of the resulted trajectories (dcd files) significantly, we removed every second frame as well as water molecules and ions using CatCDC [46]. After superposing the last 500 of the remaining 10000 frames, we calculated an average representative structure of S1-2α7-lig at the end of each 20 ns NVT simulation. This average structure has been further energy minimized with all atoms free to move. The same procedure was applied to the ligand-free S1-2α7 complex. Analysis of the final complexes for residue composition of the ligand-binding pocket, polar protein-ligand interactions, ligand binding affinities in each ligand complex was performed by employing Vina and binding affinity of S1 to 2α7 (final models) calculated by the PRODIGY server [46]. Root-mean-square deviation of atomic positions **(**RMSDs) between the final S1 model and the initial structure, and the corresponding shifts in the distance of the geometric centers between S1 and 2α7, were calculated by the VMD program [52].

## 3 Results

### 3.1 Sequence alignment

**Fig. 2:**
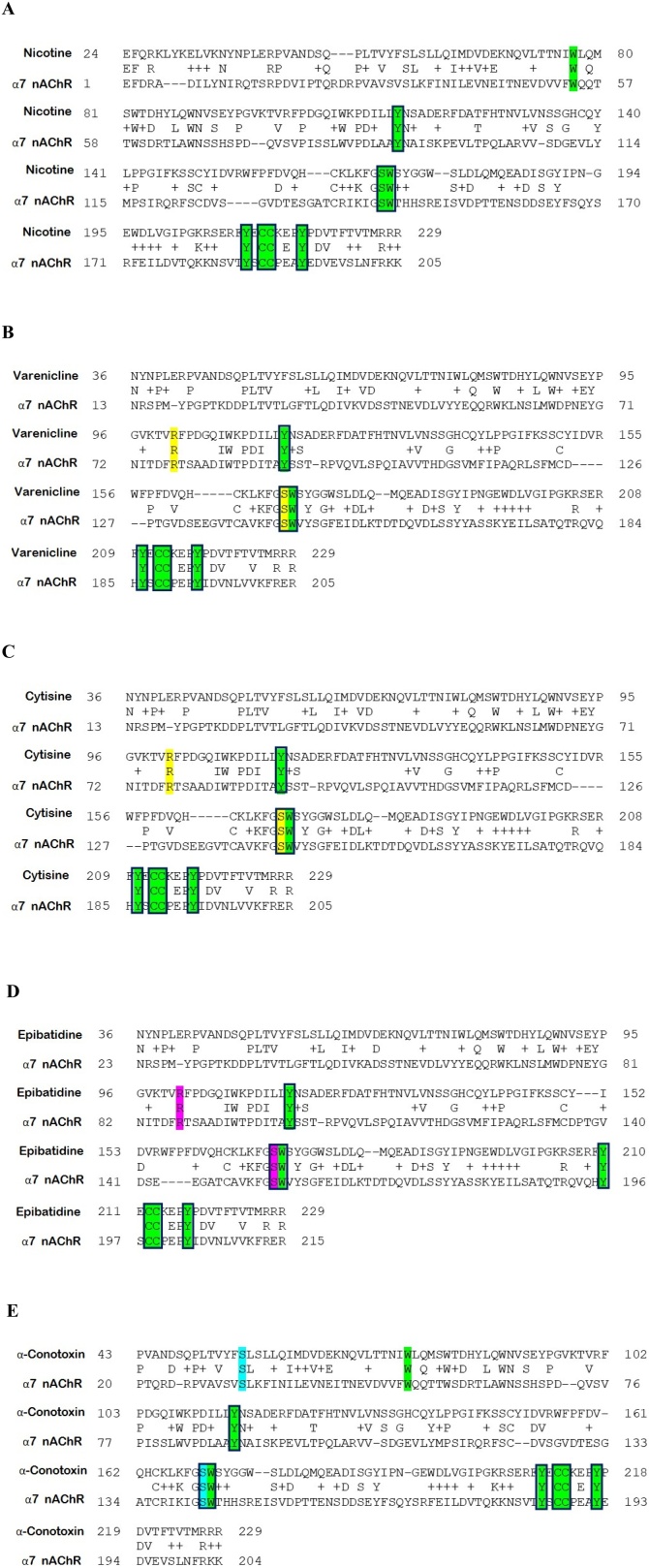
Sequence alignments of α7 nAChR (PDB: P36544) along with the respective coordinating structures. A. Nicotine (1UW6), B. Varenicline (4AFT), C. Cytisine (4BQT), D. Epibatidine (2BYN), and E.α-Conotoxin (5T90). Colored aa: conserved in human nAChR α7 subunit sequence, coordinating the respective agonist/antagonist compounds (Yellow: varenicline/cytisine, Magenta: epibatidine, Cyan: α-conotoxin, Green: nicotine). Framed aa: conserved among human nAChR α7 subunit LBD, SARS- -2 Spike glycoprotein, and AChBP.

The sequence of α7 nAChR (PDB id: 3SQ9) with all points of interaction with the respective coordinating structures of nicotine (from PDB id: 1UW6), varenicline (from PDB id: 4AFT), cytisine (from PDB id: 4BQT), epibatidine (from PDB id: 2BYN), and α-conotoxin (from PDB id: 5T90) are presented in Fig. 2. As we have previously reported [[36] Preprint], α7 nAChRs appear to possess a LBD that could harbor toxin-like sequences identified in the Receptor Binding Domain (RBD) of the SARS-CoV and SARS-CoV-2 Spike glycoproteins (A7J8L4, P0DTC2). This specific LBD comprises residues preserved in the binding site of nAChRs and shows homology across the AChBP sequences that bind the agonists, as mentioned earlier. Specifically, residues critical for the interaction between α7 nAChRs and SARS-CoV and SARS-CoV-2 Spike RBDs (^115^Y, ^171^W, ^210^Y, ^212,213^C, ^217^Y) are present in the binding motif of nicotine as well as the rest of the studied compounds. These common residues involved in nicotine and other ligand coordination are preserved in sequence and 3D location, contributing to their binding with the receptor. The binding motifs of varenicline and cytisine consist of ^91^Y, ^145^W, ^186^Y, ^188,189^C, and ^193^Y. Almost identical binding motif pattern is found for epibatidine (^93^Y, ^147^W, ^188^Y, ^190,191^C, ^195^Y), α-conotoxin (^91^Y, ^147^W, ^190,191^C, ^195^Y), and nicotine (^89^Y, ^143^W, ^185^Y, ^187,188^C, ^192^Y), respectively.

### 3.2 Interaction of SARS-CoV-2 Spike with the ECD of AChBP and cholinergic agonists

We have previously described the potential interaction between SARS-CoV-2 Spike glycoprotein (aa 381–386) and α7 nAChR ECD (aa 189–192) in a region that forms the center of the nAChR toxin-binding site. Therefore, we propose the potential competition among the various compounds and the SARS-CoV-2 Spike glycoprotein for the LBD of AChBP. For our experimental purposes (docking simulations), we used the AChBP, as all previously reported X-ray structures of nAChR agonists were mainly initially revealed with AChBP. Also, homopentamers can be formed by AChBP with pharmacology that strongly resembles those of the α7 nAChRs. As such, AChBP is considered an ideal research model for studying the LBD of the nAChRs.

**Fig. 3:**
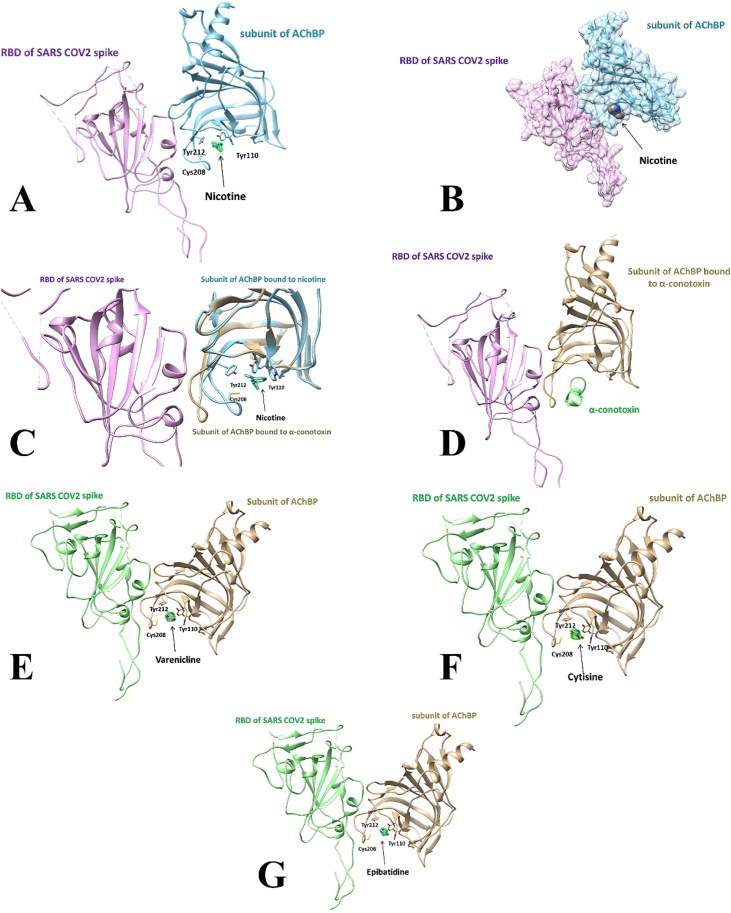
Protein complexes of the AChBP, RBD of SARS-CoV-2 Spike glycoprotein, and cholinergic agonists. A. Nicotine, B. Nicotine with a surface presentation, C. Nicotine and α7 nAChR shown in both bound and unbound (*i.e.*, apo) structure, D. α-Conotoxin, E. Varenicline, F. Cytisine, G. Epibatidine.

The complex of AChBP with nicotine and RBD of SARS CoV-2 Spike glycoprotein bound on superimposition is shown in Fig. 3A. Nicotine occupies the same volume inside the binding loop that is formed into the AChBP, with three characteristic residues coordinating it: ^110^Y, ^208^C, and ^212^Y as the RBD of SARS CoV-2. The nicotine occupied binding pocket hinders RBD of SARS CoV-2 Spike-AChBP complex formation, making binding less likely. The surface presentation of the AChBP bound to nicotine, complexed to the RBD of SARS CoV-2 Spike, is depicted in Fig. 3B. Nicotine appears inside the binding pocket of AChBP, potentially impeding SARS CoV-2 Spike from reaching the coordinating region. In Fig. 3C, the structural differences between the AChBP bound to nicotine *via* its binding loop, and the apo structure of the AChBP (*i.e.*, unbound structure) are presented.

The complex of AChBP, SARS CoV-2 Spike RBD, and α-conotoxin is shown in Fig. 3D. This cholinergic antagonist holds the LBD of AChBP inside the cavity that is formed around it. Likewise, the complex of the AChBP, RBD of SARS CoV-2 Spike glycoprotein, and varenicline is demonstrated in Fig. 3E. Again, the same pocket is formed, comprised of the same coordinating residues. Varenicline is engulfed in these amino acids and promotes a steric hindrance of the Spike glycoprotein's attachment. Fig. 3F portrays the AChBP, cytisine, and RBD of the SARS CoV-2 Spike. Cytisine is buried in the same binding site, utilizing ^110^Y, ^208^ C, and ^212^Y as coordinating residues, depriving Spike glycoprotein of the compatible motif for its binding. Fig. 3G illustrates the binding of epibatidine and AChBP in the presence of the RBD of SARS CoV-2 Spike glycoprotein. Epibatidine is located close to the residues mentioned above, inside a pocket, and weakens Spike's protein ability to interact with the locus of interest. Overall, in all cases tested, the agonists/antagonists cause a change in the conformation of AChBP, resulting in hindering the SARS CoV-2 Spike glycoprotein interaction with the nAChRs LBD.

The HADDOCK driven calculated data of the complexes formed between SARS CoV-2 Spike glycoprotein and the AChBP coupled to each of the agonists/antagonists used are recorded in Table 1. The structural and thermodynamic data represent a strong affinity of the agonists/antagonists for the AChBP, potentially interfering with the SARS-CoV-2 binding to AChBP. Also, the interaction interface of SARS-CoV-2 and AChBP, upon binding of either of the tested agonists to the latter, is partly limited, indicating their potential steric hindering action. This fact is also supported by observing the lower intermolecular contacts (*i.e.*, polar, charged, apolar) of SASR-CoV-2 and AChBP upon agonist's binding. The competitive antagonist, α-conotoxin, displays a similar effect but to a lesser degree than the other agonists mentioned above.

**Table 1:** Haddock parameters of SARS-CoV-2 Spike with ECD of AChBP (model of α7 AChRs) and potential nAChR agonists.

Parameter	α-Conotoxin	Nicotine/Cytisine/Epibatidine/Varenicline
ΔG (kcal mol^−1^)	−9.8	−9.2
Kd (Molar) at 25.0 ℃	6.6E-08	1.7E-07
Buried Surface Area	1994.1 +/− 59.7	1656.9 +/− 89.3
Van der Waals (Electrostatic) energy (kcal mol^−1^)	−70.0 +/− 6.1	−50.8 +/− 3.4
ICs charged-charged:	1	0
ICs charged-polar	12	8
ICs charged-apolar	10	8
ICs polar-polar	6	3
ICs polar-apolar	17	13
ICs apolar-apolar	18	6

### 3.3 Interference of the SARS-CoV-2 Spike – nAChR-α7 dimer complex by cholinergic agonists

**Fig. 4:**
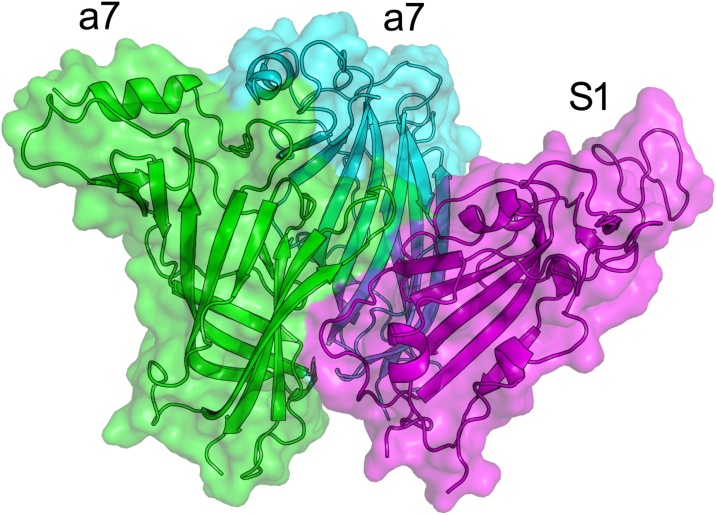
Model of S1 to nAChR-α7 dimer interaction after 20 ns MDS. Chain F (S1) in magenta, chain A (a7) in green, and chain B (a7) in cyan. Figure generated with PyMol [49].

**Fig. 5:**
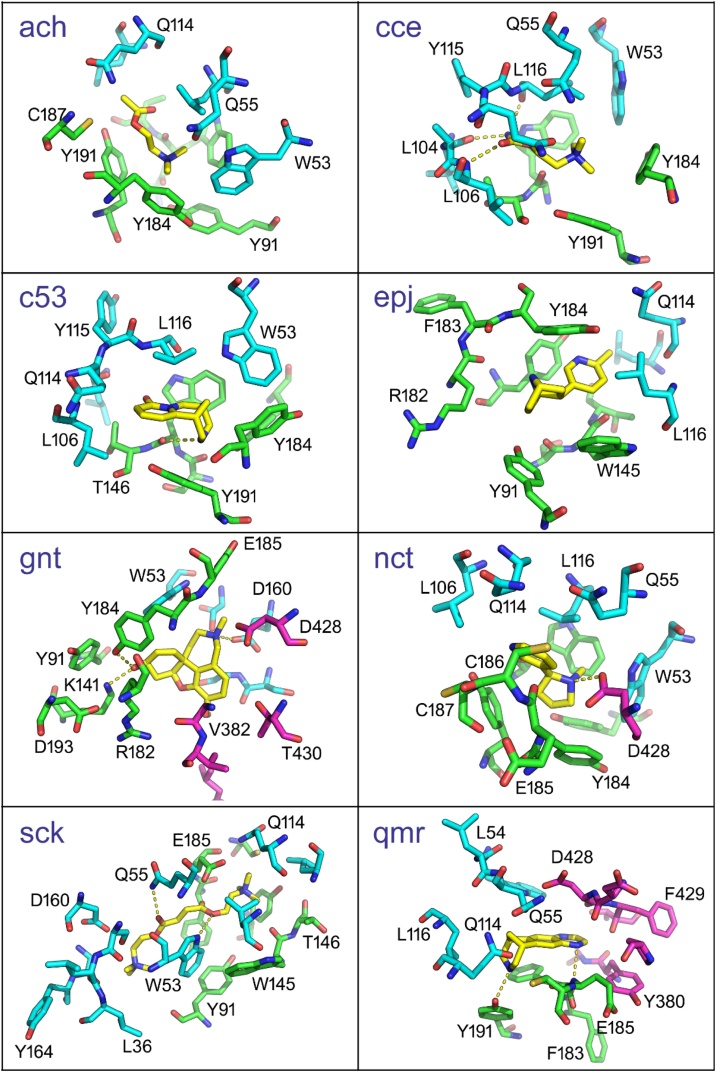
The local ligand environment in the S1-nAChR-α7-dimer ligand complexes, one panel for each ligand. Ligands are shown in yellow. Residues of the α7 chains A and B are in green and cyan, respectively, while S1 residues are shown in magenta. Yellow dots represent polar interactions.

The central question in the present study is how the binding of ligands (Table 2) to the α7 dimer could affect the binding affinity of the S1 to α7 nAChR. According to the procedure described above, the respective interactions provided models for the complex S1-nAChR-α7 with each ligand. From these models, amino acid residues at a distance ≤ 5 Å from the ligand were considered to comprise the binding pocket (see below). In this study, we have not included any crystallographically-determined water molecules within the above distance condition. The overall interaction network is reflected in the final model (after MDS) shown in Fig. 4. As expected, each ligand has shaped its interaction network to a different extent. Ligands ach, c5e, epj, and sck do not interact with the putatively bound S1. The rest of the ligands (cce, gnt, nct, qmr) all interact with ^428^D and different S1 amino acid residues (Table 2). The protein-ligand interactions in chains A and B of the α7dimer are similar. In particular, ^91^Y, ^144^S, ^145^W, ^146^T, ^184^Y, ^187^C, ^191^Y of chain A are found to interact with at least seven ligands, while chain B shows more variation, with only ^53^W, ^114^Q, ^116^L being in common in seven ligand binding-pockets. Protein-ligand polar interactions are presented in Table 3, and the ligand-binding environment is shown in Fig. 5. Interestingly, only nicotine forms a polar interaction with S1 (chain F). Quantitative characterization of the S1- nAChR-a7-dimer interactions with ligands can be found in Table 4. Here, we present several metrics to characterize the effect of the ligands' presence after 20 ns MDS. According to these metrics, ligands affect the structure of S1 as depicted by the RMSD values, as well as its relative position to the α7 dimer (S1 shift). The largest effect on these metrics is caused by the docking of succinylcholine with an S1 shift of 6.0 Å and an RMSD value of 10.08 Å. On the other hand, the lowest S1 shift is triggered by the docking of varenicline (0.051 Å) and the lowest RMSD by the docking of cytisine (2.96 Å), which are values close to or smaller than that observed in a simulation without any ligand (last row in Table 4) and this can be considered as the null effect level. Moreover, ligands are found to affect the affinity of the S1 to α7 dimer interaction as provided by the PRODIGY server [53]. Here, the most stable S1 to nAChR-α7-dimer interaction is in the presence of varenicline with a ΔG = -13.1 kcal/mol, while the least stable is that with galanthamine (ΔG = -8.0 kcal/mol). In the absence of ligands, the corresponding ΔG value is -9.7 kcal/mol. Finally, the most stable interaction of S1-2α7 to the ligand is found with varenicline (ΔG = -8.7 kcal/mol), while the least stable with acetylcholine and carbamylcholine (ΔG = -4.0 kcal/mol). As might be expected, these values show a correlation not to be ignored. Pearson correlation coefficient between S1 shift and ΔG of S1 to nAChR-α7-dimer is 0.7791, while between S1 shift and RMSD is 0.8530, and between ΔG of S1 to nAChR-α7-dimer and S1-2a to lig is 0.7713.

**Table 2:** Composition of the 5 Å binding pockets.

Ligand	S1	α7 (chain A)	α7 (chain B)
Acetylcholine (ach)		Y91, S144, W145, T146, R182, Y184, C187, Y191	W53, Q55, S77, L106, Q114, L116
Carbamylcholine (cce)	D428	Y91, S144, W145, T146, Y184, E185, C186, C187, Y191	W53, Q55, P102, L104, A105, L106, Q114, Y115, L116, P117
Cytisine (c5e)		A89, Y91, S144, W145, T146, Y184, E185, C186, C187, Y191	W53, L104, A105, L106, Q114, Y115, L116
Epibatidine (epj)		A89, Y91, S144, W145, T146, Y184, E185, C186, C187, Y191	W53, L104, A105, L106, Q114, Y115, L116
Galantamine (gnt)	G381, V382, L387, L390, D428, T430, L517	Y91, K141, W145, R182, Y184, E185, D193	S34, L36, W53, D160, S162, G163, Y164, I165
Nicotine (nct)	D428	Y91, S144, W145, T146, R182, Y184, E185, C186, C187, Y191	W53, L54, Q55, L106, Q114, L116
Succinylcholine (sck)		Y91, N92, S144, W145, T146, Y184, E185, C186, C187, Y191	S34, L35, L36, W53, Q55, L106, Q114, L116, D160, G163, Y164
Varenicline (qmr)	Y380, G381, P412, D427, D428, F429, T430	W145, F183, Y184, E185, C186, C187, Y191	W53, L54, Q55, M56, Q114, Y115, L116

**Table 3:** Protein-ligand polar interactions. Chains are shown in parentheses.

	Protein atom	Ligand atom
Acetylcholine (ach)	No hydrogen bonds detected	
Carbamylcholine (cce)	Leu104 O (B)	N6
	Leu106 N (B)	O7
	Leu116 O (B)	N6
Cytisine (c5e)	Trp145 O (A)	N1
Epibatidine (epj)	No hydrogen bonds detected	
Galantamine (gnt)	Tyr184 OH (A)	O18
	Lys141 NZ (A)	O18
	Asp160 OD1 (D)	N10
Nicotine (nct)	Asp428 OD2 (F)	N2
Succinylcholine (sck)	Trp53 O (B)	O14
	Gln55 NE2 (B)	O7
Varenicline (qmr)	Tyr191 OH (A)	NO2
	Glu185 N (A)	N13

**Table 4:** Flexible docking results of various ligands to the S1-nAChR-α7-dimer complex (S1 bound to an α7 dimer) before and after MDS.

		Docking	After 20 ns MDS			
Ligand	PDB code	Prot-Lig ΔG (Vina) [kcal/mol]	*S1 shift [Å]	#S1 RMSD [Å]	Prot-Lig ΔG (Vina) [kcal/mol]	S1−2α7 ΔG (Prodigy) [kcal/mol]
Acetylcholine (ach)	3WIP	Model 1 −3.7	0.977	5.617	−4.0	−9.6
Carbamylcholine (cce)	1UV6	Model 1 −3.8	1.756	4.569	−4.0	−10.4
Cytisine (c5e)	4BQT	Model 8 −7.8	0.611	2.958	−8.5	−12.4
Epibatidine (epj)	3SQ6	Model 3 −7.5	0.244	4.746	−7.4	−11.5
Galantamine (gnt)	2PH9	Model 1 −10.9	3.028	8.568	−6.0	−8.0
Nicotine (nct)	1UW6	Model 6 −5.9	0.285	5.603	−6.9	−12.6
Succinylcholine (sck)	2HA2	Model 2 −5.6	5.979	10.083	−3.7	−8.3
Varenicline (qmr)	4AFT	Model 8 −7.2	0.0512	3.971	−8.7	−13.1
S1-2α7 complex, no ligand			1.075	3.062		−9.7

* Shift of S1 geometrical center from 2α7 geometrical center after 20 ns MDS.

# RMSD of S1 after MDS relative to its starting position without correction for translation and rotation. That is S1 RMSD includes conformational as well as positional changes.

## 4 Discussion

Smoking is a well-identified cause of increased vulnerability and gravity in the respiratory tract [11]. Severe COVID-19 outcomes were probably because of underlying breathing diseases, particularly COPD and cigarette smoking [54,55]. Since COVID-19 is a contagious condition that mostly impacts the lungs and harms the lungs' function, smoking can make it more difficult for the body to fight against coronaviruses and other diseases. Tobacco is also a vital risk for non-communicable diseases such as cardiovascular, cancer, and diabetic issues, which raise the threat of significant COVID 19 condition. On the other hand, if used in other ways, nicotine, the addicting component of cigarettes, might be safe, as well as there is some biological plausibility regarding the possible function of pure nicotine in infection with COVID-19 [64,65].

Based on clinical observations on smoking and COVID-19 hospitalization and our *in silico* findings, we have built a hypothesis that SARS-CoV-2 Spike glycoprotein, bearing a "toxin-like" sequence in its RBD, could bind to the toxin-binding domain of the α-subunit of the nAChRs. This binding might produce several adverse effects by dysregulating the NCS, in which α7 nAChRs are principally involved. Dysfunction of the cholinergic anti-inflammatory pathway may lead to a cytokine storm and the immune response's failure to return to homeostasis. Clinical manifestations of COVID-19 might also be explained by cholinergic dysfunction [11]. If proven *in vivo*, these findings could have important therapeutic implications, as nicotine may partially reverse this binding, while other compounds acting as full or partial agonists to nAChRS may also compete for binding with SARS-CoV-2 Spike glycoprotein.

We have previously described the molecular complexes of human α7 nAChR to both SARS-CoV and SARS-CoV-2 Spike glycoproteins, either in their open or closed conformation [[36], preprint]. We have observed that a significant portion of the "toxin-like" sequence in SARS-CoV and SARS-CoV-2 Spike can interact with the toxin binding sites of human α7 nAChR in the nM range, which is comparable with experimental supported Kds of well-known enzymatic interacting partners that produce stable protein complexes [56]. The main intermolecular contact clusters at the interface for the complexes between SARS-CoV-2 Spike glycoproteins and the LBD of the human α7 nAChR involve sequence regions ^383^S-^388^C and ^207^E-^217^Y. Similarly, the SARS-CoV-2 conserved residues ^384^P and ^385^T, are in close contact with ^214^K and ^209^ F of the α7 AChR subunit.

In this work, we are broadening our previous findings by presenting the most common nAChRs agonists' molecular docking with the homologous to nAChRs, the AChBP, and the S1-nAChR-α7 complex. AChBP secreted from glial cells in the central nervous system of the seawater snail modulates synaptic transmission. It lacks both the transmembrane domains and the intracellular loops typical of the nAChRs. However, it is found to form homopentamers with pharmacology resembling that of α7 nAChRs. As such, AChBP is considered an ideal model for studying the LBD of the nAChRs. It is possible that cholinergic agonists/antagonists (*i.e.*, nicotine, cystine, epibatidine, and varenicline) could impede the interaction between human nAChRs and SARS-CoV Spike RBD. The coordination of nicotine and the rest agonists/antagonist is driven by a highly conserved group of amino acids in their respective structures, identically recognized by the LBD located on the nAChRs structure.

Interestingly, residues critical for the interaction between α7 nAChRs and SARS-CoV and SARS-CoV-2 Spike RBDs (^115^Y, ^171^W, ^210^Y, ^212,213^C, ^217^Y) are present in the binding motif of nicotine as well as the rest of the composites. These common residues hold the same position, are conserved in their sequences, and coordinate to nicotine and the rest of the compounds, whereas other adjacent residues also contribute to their binding. The α7 nAChRs and S1 interaction is significantly disturbed by the binding of each of the seven ligands since seven residues of chain A, and three of chain B were found to interact with all seven ligands reducing thus the α7 surface availability for interaction. From the seven ligands studied, the binding of galanthamine or succinylcholine to α7 seems to destabilize the S1-2α7 complex formation. Interestingly, only nicotine forms an additional polar interaction with S1, indicating that this ligand may have the potential to influence more effectively the α7 nAChRs and S1 interaction. Among the seven ligands studied, succinylcholine seems to trigger the largest positional (including conformational) changes to S1, while cytisine does not cause any conformational change upon binding. However, the latter should be investigated in-depth to explore their possible role in the LBD of nAChRs, as far as interaction with SARS-CoV and SARS-CoV-2 Spike RBDs is concerned.

NCS is implicated in a variety of other mediated human pathologies. Indeed, the anti-inflammatory effects of nicotine on obesity and ulcerative colitis have already been documented. Lakhan and Kirchgessner [57] reported that smokers have a lower occurrence of certain inflammatory diseases, including ulcerative colitis, and that the protective effect includes the activation of the NCS, which requires the existence of α7 nAChR on immune cells. Inflammation resulting in epithelial barrier disruption is a hallmark of inflammatory bowel disease, and nicotine appears to be protective in ulcerative colitis [57]. Additionally, the exploitation of the cholinergic anti-inflammatory pathway to treat epithelial inflammatory diseases has also been previously investigated. Nevertheless, its efficacy as a treatment for inflammatory bowel diseases remains controversial [58].

Similarly, NCS is reported to be also involved in several viral-induced human pathologies. Cheng and Li-Sha have shown the dose-related effects of nicotine in a coxsackievirus B3 murine myocarditis model [59,60]. Specifically, it was found that nicotine reduced the severity of viral myocarditis by inhibiting the production of proinflammatory cytokines. They found that α7 nAChR activation increases STAT3 phosphorylation, decreases TNF-α and IL-6 expressions, and ultimately reduces viral myocarditis, indicating that α7 nAChR agonists could be a promising new strategy for patients with viral myocarditis. Also, a recent case study documented the post-infectious onset of myasthenia gravis in a COVID-19 patient, indicating that the SARS-CoV-2 virus could be an etiological agent in this case [61]. It is well-established that myasthenia gravis is associated with the presence of AChR antibodies, although it is unclear how this could be linked to the interactions presented herein.

## 5 Conclusions

Our findings herein support our hypothesis that SARS-CoV-2 could interact with nAChRs triggering NCS dysregulation and that partial or full agonists of AChRs may be an intriguing therapeutic approach for the COVID-19s' pandemic. Cholinergic agonists may inhibit an interaction between SARS-CoV-2 and nAChRs. In that context, we suggest that the potential therapeutic effects of nicotine or other cholinergic agonists, including those examined herein, to combat COVID-19 pandemic infection should be further investigated *in vitro* and *in vivo* by experimental validation studies. For example, Saturation Transfer Difference (STD) NMR spectroscopy could be performed to screen potential ligands bound to recombinantly expressed α7 nAChRs and map their binding site properties to determine the dissociation constants. These types of experiments are sensitive enough to detect ligand binding at protein concentration levels frequently found in cells (pM–nM). This approach could relieve the need for high protein yields from expression systems, making bacterial expression systems unnecessary.

## Author’s statement

The authors hereby declare:

• all authors have seen and approved the final version of the manuscript being submitted. They warrant that the article is the authors' original work, hasn't received prior publication and isn't under consideration for publication elsewhere.
• Socrates J. Tzartos, Elias Eliopoulos, Konstantinos Poulas and Konstantinos Farsalinos are listed as inventors on pending patent application for cholinergic agonists and anti-SPIKE antibodies.
• this research did not receive any specific grant from funding agencies in the public, commercial, or not-for-profit sectors.

## Funding statement

This research did not receive any specific grant from funding agencies in the public, commercial, or not-for-profit sectors.

## CRediT authorship contribution statement

**Nikolaos Alexandris:** Methodology, Investigation, Software, Writing - original draft, Writing - review & editing. **George Lagoumintzis:** Methodology, Investigation, Software, Writing - original draft, Writing - review & editing. **Christos T. Chasapis:** Methodology, Investigation, Software, Writing - review & editing. **Demetres D. Leonidas:** Methodology, Software, Writing - review & editing. **Georgios E. Papadopoulos:** Methodology, Software. **Socrates J. Tzartos:** Methodology, Writing - review & editing. **Aristidis Tsatsakis:** Methodology, Writing - review & editing. **Elias Eliopoulos:** Methodology, Software, Writing - review & editing. **Konstantinos Poulas:** Writing - review & editing, Conceptualization, Supervision. **Konstantinos Farsalinos:** Writing - original draft, Supervision.

## Declaration of Competing Interest

The authors report no declarations of interest.
